# Socio-Structural Barriers, Protective Factors, and HIV Risk Among Central-Asian Female Migrants in Moscow

**DOI:** 10.5195/cajgh.2013.31

**Published:** 2013-07-16

**Authors:** Christopher Zabrocki, Stevan Weine, Stephanie Chen, Ivana Brajkovic, Mahbat Bahromov, Sana Loue, Jonbek Jonbekov, Farzona Shoakova

**Affiliations:** 1 The University of Illinois at Chicago, Chicago, Illinois, USA; 2 Prisma Research Center, Dushanbe, Tajikistan; 3 Case Western Reserve University, Cleveland, Ohio, USA; 4 Prisma Research Center, Moscow, Russia

**Keywords:** HIV/AIDS, Women’s Health, Central Asia, Risk Perception

## Abstract

**Objective:**

This study aimed to build formative knowledge on socio-structural barriers, protective factors, and HIV sexual risk amongst Central-Asian female migrants in Moscow.

**Methods:**

Data collection included ethnographic interviews in Moscow with a purposive sample of 30 unmarried female migrants, 15 from Kyrgyzstan and 15 from Tajikistan.

**Results:**

Study participants reported difficulties with acquiring documents for legal status, financial insecurity, discrimination, sexual harassment, and lack of support. Based on analysis of the cases, one pathway linked lack of legal documentation and instrumental support with elevated sexual risk. Another pathways linked traditional cultural attitudes with both no and moderate sexual risk.

**Conclusion:**

Future HIV prevention efforts with Central Asian female migrants in Moscow should be multilevel and include: increasing HIV and prevention knowledge and skills, promoting condom use with regular partners, identifying and supporting cultural attitudes that protect against HIV sexual risk behaviors, facilitating legal status, building community support, and increasing economic options.

Women account for increasing numbers of labor migrants globally and approximately 15 to 30% of migrants to Russia.^1^,^2^ Between 2004 and 2008, approximately 800,000 migrants traveled to Moscow from Kyrgyzstan, and another 1.5 million from Tajikistan.^3^ Due to the high prevalence of HIV in Moscow and the sexual practices of male migrants, public health experts are concerned that migrants could be a bridge for HIV transmission to their lower HIV prevalence home countries, including Tajikistan and Kyrgyzstan.^4^,^5^

Female migrants may be at increased risk for acquiring HIV infection in multiple ways. Away from their home countries’ traditional cultures that prohibit women from engaging in non-marital sexual relations, female migrants may be more sexually active with more partners.^6^–^9^ In Moscow female migrants face greater difficulties with legal status than male migrants.^10^,^11^ These difficulties can increase their vulnerability to exploitation, reduce their access to services, and lead to higher STI and HIV.^12^–^14^ Illegal status may confine female migrants to unregistered and low-wage positions.^15^ To survive, women often turn to formal sex work or to transactional sex, resulting in greater sexual risk for HIV.^16^–^18^ Female migrants from developing countries often have limited HIV knowledge, may associate condoms with promiscuity, and may avoid condom use with regular sexual partners.^19^–^24^

On the other hand, home country religion, cultural values of abstinence, and having friends not engaged in sexual risk behavior may be protective factors for HIV risk.^25^–^27^ Respect for parents’ beliefs and values and having plans for the future have been found to be protective against pre-marital sex.^28^

The WHO has shifted the emphasis of preventative health care with migrants from a focus on individual behavior to considering the social determinants of health.^29^ However, HIV risk among the growing number of female labor migrants globally, is presently under-investigated.^30^ There is a need for scientific investigations of female migrants that build knowledge on the social determinants of health and that could help to inform the development of HIV prevention for female labor migrants.

This ethnographic study sought to learn about Central Asian female migrants’: 1) working and living conditions in Moscow; 2) HIV/AIDS knowledge, attitudes and behaviors; and 3) HIV risk in relation to socio-structural barriers and protective factors. Lastly, the study aimed to consider implications for future programs, policies, and research that address the social determinants of health among female migrants.

## Methods

### Sampling and Recruiting

The study purposively sampled thirty female migrants currently living in Moscow. This sampling strategy maximized diversity on three axes: (1) age; (2) education; (3) employment in Moscow. Fifteen were from Kyrgyzstan and fifteen were from Tajikistan. Eligibility required that the women be: (1) unmarried; (2) 18–35 years old; (3) in Moscow for the first time; (4) living in Moscow less than 1 year; (5) from Tajikistan or Kyrgyzstan; (6) able to give informed consent. In Moscow, Tajik female migrants were recruited through several Tajik diaspora organizations and Kyrgyz female migrants through the Kyrgyz diaspora center. After describing the study, oral informed consent was obtained, as approved by the IRBs of the University of Illinois at Chicago, the Tajik Ministry of Health, the Russian Academy of Arts and Sciences, and Case Western Reserve University.

### Data Collection

With each migrant we conducted a single minimally structured interview in English with interpretation in either Tajik or Russian by bilingual team members.^31^ The interviews lasted between 60 and 150 minutes and were conducted in convenient locations for the participants, such as apartments, parks, cafes, or the research team’s office. Participants were paid $20. These open-ended interviews focused on the women’s: (1) daily lives; (2) experiences with migration and life in Moscow; (3) home country lives and family; (4) social support and network in Moscow; (5) HIV/AIDS knowledge, attitudes, behaviors, and risk and prevention skills; and (6) access to healthcare and HIV testing. Demographic information was also gathered (Table 1). All interviews were audiotaped and transcribed into English. The initial study questions were refined through an iterative process of data collection and preliminary data analysis that followed standardized qualitative methods.

### Data Analysis

The study utilized a grounded theory approach to qualitative analysis and Atlas/ti computer software.^32^,^33^ After establishing coder reliability, all transcripts were coded. Key variables, categories, processes, and theoretical claims emerged through pattern coding and memoing.^34^ The findings were reviewed by the entire team to check for contrary evidence.

The determination of instrumental support and legal status was done by consensus rating of the entire research team based upon both established definitions and knowledge gained from the female migrants. For instrumental support we used the Tilden and Weinhart definition of the provision of tangible goods, services or aid.^35^ A female migrant was considered to have instrumental support if she identified someone who could provide her with food, money, or employment. Regarding legal status, we learned from official sources and from the female migrants that it was important to consider migration documents, work permits, and registration.^36^ The female migrant was considered to have legal status only if she reported having acquired all these documents in Moscow. Regarding HIV sexual risk behaviors, the participants were classified into three sexual risk categories based on their reported current behavior as migrants: no risk (not currently sexually active), moderate risk (currently sexually active, monogamous), and elevated risk (currently sexually active, transactional sex or sex work).

## Results

### Surviving in Moscow

All of the women reported that the primary reason for leaving home and coming to Moscow was to earn money. Although some female migrants came with education and skills, most could only find work that was far below their skill level. One reported, “To work here within your profession is very difficult. I am educated in economics and have worked in a bank as a cashier in Kyrgyzstan. It is impossible to work in such jobs here” (Kyrgyz, 23A).

Most who were employed reported being paid too little and intermittently or not at all. One Tajik migrant reported, “For almost one year I’ve worked in Moscow, but I haven’t seen any money from employers. Every time I have worked; the employer throws me out” (Tajik, 31).

Others told of not being able to find work at all due to discrimination. “Here I went to several places and they said you should be a citizen of Russia, and besides, we take only Slavic appearance” (Kyrgyz, 35). Without work or money, some women had to live on the street. One reported, “I took these clothes from the trash dump, because I didn’t have any money. I was suffering a lot and I’m suffering still, I have slept in the streets and in entrances, because there are not any good jobs and no salaries” (Tajik, 31).

Many participants reported that workplace sexual harassment was commonplace. “Some people would touch me and pinch my butt when walking through. I worked there almost eight months” (Tajik, 19A). Some reported employers withholding salaries unless migrants performed sexual favors. “In some places the employer man offered me to have sex with him, just after that he would pay me my salary, but I refused, I didn’t want to do that” (Tajik, 31). When she refused, she became subject to further harassment by her employer: “For two days I worked there and everything was fine, but the other day the boss got drunk and wanted me to have sex with him. I didn’t agree and he started to beat me.” Another said, “He wants me to have sex with him, but I don’t want to have such a sexual partner, therefore he always tries to hurt me and behaves badly towards me” (Tajik, 32).

Many female migrants reported encountering deception, greed, and selfishness amongst other female and male migrants in Moscow. One said, “You care about others there, and here you only care about yourself. You become an egoist, you stop caring about others” (Kyrgyz, 23A). Another said, “Moscow makes people lie” (Kyrgyz, 26). As a consequence they learned to think more negatively about others and themselves. “I think that everybody is going to cheat us again” (Tajik, 31). With little community support, female migrants rely principally on themselves, “Nobody except yourself can help you; you are alone in this big city” (Kyrgyz, 27).

### Not Worried about HIV

Although several female migrants reported no knowledge of HIV, most knew something about AIDS, HIV transmission, and HIV prevention. Many stated that HIV was present in Russians and marginalized groups, such as sex workers, partiers, and the rich, but they did not believe that HIV/AIDS was as prevalent in persons from their home country. “I didn’t hear that this disease was among Tajik women, but I know that it is spread among Russian women” (Tajik, 31). Another stated, “The rich guy that spends money for fun is definitely infected. I am dating an ordinary simple man who is not infected” (Kyrgyz, 28). Another said, “I think all the street girls who work in public houses or in the street are all infected with STIs and no man should have sex with them” (Tajik, 19A). Female migrants reported knowing that condoms could prevent HIV transmission; one said, “The big reason is to protect from STIs, HIV and other infections that can be transmitted sexually” (Tajik, 26). HIV infection was regarded as a death sentence and as a social stigma. One migrant said, “The man that is HIV+ is just a dead man. He is just a living body. He only has the end and nothing else” (Kyrgyz, 28).

### Current HIV Sexual Risk Behaviors as Migrants

#### Not Currently Sexually Active (No Risk)

Eighteen (60%) female migrants reported no current sexual activity. Some of them were dedicated to lost partners: “The passion and the love I had with my marriage I lost, I had a good life with my husband, but I lost him long ago… but now my son is already a big boy and I have been without a husband for a long time and [being single] is normal for me” (Tajik, 35). Others wanted to remain a virgin until marriage. “We are Muslims and we are not allowed to have sex before marriage” (Tajik, 25). Still others reported no sexual desire or an inability to find a suitable partner, “I haven’t found such a man that I could love yet” (Tajik, 34). Some told of being uninterested in relationships, “If you get married you have to do what your husband and his family says. I want to live independent and communicate with my friends when I want and where I want” (Kyrgyz, 23A). Others prioritized different goals. “I do not want anyone at all because if you have someone; you will not work” (Tajik, 27A). Regarding condoms, one woman said they were not for “love partners,” and she did not plan to use them when in a relationship, “Those girls who do sex before marriage, I’ve heard that they use condoms. I think only ‘street girls’ can use condoms; not normal girls. When you love your man, why should you use it?” (Tajik, 19B). Based upon their reported current sexual behavior, as migrants these women were not considered to be at HIV risk.

#### Currently Sexually Active, Monogamous (Moderate Risk)

Eight (27%) female migrants reported that they were sexually active, but monogamous with one partner in Moscow, usually another migrant. These women reported that they were planning eventually to marry their sexual partner, “My attitude towards sex is that if you trust and love your boyfriend it is a benefit because the body requires it. When you are with one man, it is good.” (Tajik, 19A). She continued saying, “Many people have sex with condoms, but I think condoms are a disrespect to your body. Why should I use a condom if I am having sex with my love.” Another said, “I do not feel good when he uses condoms. For me, when he uses condoms, it is like he keeps away from me. I feel like he uses me if he uses a condom. Like, he would use condoms with any girl” (Tajik, 22). Although the participants did not report suspecting that their partners had sexual relations with other women or sex workers, our knowledge suggests that this is very likely.^11^ although these women are currently monogamous, they are considered to be at moderate HIV risk.

#### Currently Sexually Active, Transactional Sex or Sex Work (Elevated Risk)

Four (13%) of the female migrants were involved in either transactional sex (n=2) or sex work (n=2). The women who performed transactional sex reported needing money or a place to stay. “In Russia, it is normal to have sex for money, it is not shameful. Though for us it is shameful to ask another for help this way” (Tajik, 33B). Their partners sometimes included married migrant men and drug users. Both of the women engaged in transactional sex reported always using condoms, “When I have sex I use condoms, and thus I protect myself” (Tajik, 33A). The sex workers reported working independently and saw mostly Russian men. Both of the migrants who did sex work reported inconsistent condom use. One sex worker always used a condom with her new clients, “They say they will pay more if I refuse to use a condom, but I do not pay attention to them. I say that I will use a condom. I understand that if I catch a disease, this money will not be enough to get cured” (Kyrgyz, 30). Another woman who did sex work said, “Sometimes I use condoms. If I meet with a guy for the first time then I use it, but with the familiar guys I do not. I can trust the familiar ones” (Tajik, 33B). Due to their many concurrent sexual partners, and their inconsistent use of condoms, these migrants are considered at elevated HIV risk.

#### Roles of Socio-Structural Barriers and Protective Factors

Qualitative findings were used to characterize the possible roles of socio-structural barriers and protective factors.

#### Socio-Structural Barriers as Facilitators of Sexual Risk

Some female migrants lacked the proper documentation for migrants living and working in Moscow. They most often mentioned problems obtaining work permits and Moscow registration, stating they could not afford to pay twelve thousand rubles (approximately $400) for a work permit. One said, “To find work, you should have documentation, registration, and work permission. I made a registration when I came, but it was hard to do the work permission. I found that the work permission is very expensive. I found that I have to pay for everything, and it is difficult” (Kyrgyz, 30). Another reported, “When you do all your documents then you will find a job. If you don’t you won’t find a job” (Kyrgyz, 23B). Women said that without documents, they were more likely to be arrested and detained by the police, who would expect bribes or favors, or be sexually harassed and exploited by employers, “If you have no documents, you have no freedom” (Kyrgyz, 27). Some women reported giving in to the sexual demands of their employer in return for money.

Most female migrants reported lacking family or friends who could provide them with financial, material, or other tangible support. One stated, “I don’t have close friends in Moscow, just girls whom we live together and we say each other just ‘hello’ and ‘goodbye’ and nothing more. I don’t have close friends to share something with” (Tajik, 34). Some women were recruited into sex work by female friends: “She did not tell me directly, she just invited me to her apartment. There were two men, and she introduced me to one. We talked and talked. She then said that he will help me and give me money, and he did” (Tajik, 33B).

#### Cultural Attitudes as Protective Factors Against HIV Sexual Risk**.**

Some migrants who were not involved in transactional sex or sex work expressed cultural attitudes towards sexual relations before marriage. These cultural attitudes were found in both Tajik and Kyrgyz migrants. One woman said, “I understand that at my age it is a normal time to have sex. But according to our oriental mentality, I try to protect myself from sexual contact till my wedding day” (Tajik, 27B). Another stated, “I was brought up by my parents in a way that I can’t have sexual relations with anybody until marriage. There can be anything before marriage” (Kyrgyz, 27). Those migrants who did not pursue transactional sex or sex work despite socio-structural barriers reported more traditional cultural attitudes towards sex work, health, and insecurity. Again, these cultural attitudes were also found in both Tajik and Kyrgyz migrants. Several were unwilling to do sex work because of its sinfulness. One stated, “I was offered several times but it is a great sin, therefore I refused. I don’t want to make me “haraam,” [Arabic for “forbidden”] the “haraam” money makes no sense for me, and I don’t want to make my body dirty with dirty men” (Tajik, 31). Another stated, “I don’t want to do sex work; I want to earn my salary honestly (Kyrgyz, 22).” Others reported worries over the insecurity of transactional sex. One stated: “I know it is only temporary. They will use you … kick you out …you will again be alone. I do not want such things. I am only interested in my work” (Tajik, 27A). Of course cultural attitudes would not necessarily protect female migrants against forced sex, manipulation, or trafficking.

#### Hypothesized Pathways Involving Socio-Structural Barriers and Protective Factors

**Figure 1:**
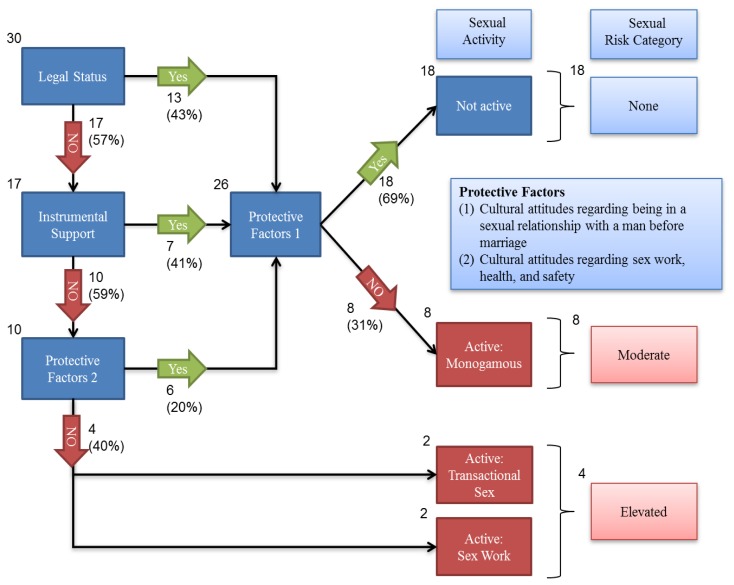
Hypothesized Pathways Involving Socio-Structural Barriers and Protective Factors

Figure 1 represents hypothesized pathways connecting socio-structural barriers, possible protective factors, current sexual activity, and level of HIV sexual risk based upon the study findings.

Regarding risks, this figure indicates a hypothesized pathway linking legal documentation and instrumental support with elevated sexual risk. Specifically, four (100%) of the women in current transactional sex or sex work had neither legal documentation nor instrumental support. In comparison, 2 of 8 (25%) currently sexually active, monogamous, and 4 of 18 (22%) not currently sexual active lacked instrumental support and legal status.

Regarding protective factors, this figure indicates how protective factors may partly explain why some female migrants exposed to socio-structural barriers exhibit none or moderate current sexual risk. Specifically, of the 20 female migrants with either legal status or instrumental support, 12 (60%) were not currently sexually active, one possible indicator of protective factors. Of those 10 female migrants lacking both legal status and instrumental support, 4 (40%) were not currently sexually active, and 2 (20%) were currently sexually active and monogamous, another possible indicator of protective factors.

## Discussion

Central Asian female migrants in Moscow reported difficulties with acquiring documents for legal status, financial insecurity, discrimination, sexual harassment, and lack of support. These difficulties are viewed as consequences of socio-structural barriers which are shaped by underlying social, economic, and political phenomena, not individual inadequacies. This study also found qualitative evidence suggesting possible associations between socio-structural barriers (e.g. lacking both legal status and instrumental support) and current elevated HIV sexual risk.

The study findings indicated that socio-structural barriers did not explain all HIV sexual risk. Some women were sexually active and monogamous; however, their regular partners were migrants and thus likely to be sexually active with multiple partners concurrently.^11^ Some women engaged in sex work or in transactional sex preferred not using condoms, despite their knowledge of HIV/AIDS and HIV prevention skills. This preference was especially true with regular clients, a pattern which has been found elsewhere.^22^

Cultural attitudes regarding sex work, health, and insecurity could be protective in female migrants who chose not to do sex work or transactional sex. To lower risk behaviors, HIV preventive interventions may try to facilitate changes in female migrants’ cultural attitudes. However, this intervention model is limited by its focus on individual level change in ideology rather than contextual changes; a limitation previously noted in U.S.-derived HIV preventive interventions.^37^

Several additional policy and program steps are warranted. Through bilateral agreements between the Russian Federation and Kyrgyzstan and Tajikistan, legal steps could be taken to improve female migrants’ ability to obtain proper documentation for legal status in Russia, thus enabling more to work and earn money through means other than sex work.

Health has been framed as a human right in multiple international human rights treaties including the Convention on Elimination of Discrimination Against Women (CEDAW), to which Russia, Tajikistan, and Kyrgyzstan are each signatories. It stipulates the health rights of labor migrants, including HIV prevention and care.^38^ This treaty requires actions from each national government to protect female migrants’ health rights through providing health and social services at three times and locations: 1) prior to migration; 2) during their stay in the receiving country; 3) upon reintegration in the sending countries. In particular, HIV preventive interventions for female migrants should be conducted in the sending countries both pre and post migration, in transit, and in the receiving country. Another priority is the establishment of community support networks in the receiving country that would provide needed instrumental, social, and emotional support for female migrants. This should include programs for income generation through cooperatives that would protect female migrants from unwilling entry into sex work or transactional sex, as well as to assist those wanting to leave sex work or cease transactional sex.^39^ These health and social service initiatives are the responsibility of the sending and receiving countries and require coordinated, joint actions.

Regarding research implications, this study produced a model linking health outcomes and social determinants that requires further investigation in order to support new program and policy initiatives for the growing global trend of female labor migrants who find themselves in high risk environments. Key research questions regarding female labor migrants include: Do socio-structural barriers predict sexual risk? What is the protective role of cultural attitudes? What are the pathways, over time, by which female migrants move into or out of sexual risk behaviors? What socio-structural barriers and cultural attitudes are potentially modifiable by multilevel interventions? How can the constraints upon community leaders, organizations, and policymakers best be managed? How can community collaborative approaches best be used to develop, implement, and evaluate new interventions and policies? Rigorous, longitudinal, mixed method studies with both purposive and probabilistic samples are needed.

This study has several limitations. First, there is possibility of misunderstanding due to interpretation. We addressed this challenge through a multi-lingual research team and ongoing review of translation. Second, the sample was not representative of the entire female migrant population in Russia, which includes women from other countries. Third, because participants were recruited through organizations, those most isolated and perhaps with greater sexual risk were not included. Fourth, because this study was cross-sectional, we could not follow women’s actual pathways regarding migration and HIV sexual risk over time. Fifth, because there sample was purposive and the cells too small, we could not test the associations statistically.

## Conclusions

This study addresses the understudied global phenomenon of the feminization of labor migration and focuses on their HIV risk. It builds knowledge regarding the unique multi-level contextual variables involving structural, social, and cultural level factors that need to be understood in developing HIV preventive intervention for sexually active female migrants. Future HIV prevention efforts with Central Asian female migrants in Moscow should focus on facilitating legal status, building community support, promoting condom use with regular partners, identifying and supporting cultural attitudes that protect against HIV sexual risk behaviors, and rigorous research testing of these hypothesized pathways.

## Figures and Tables

**Table 1:** Demographic Information

	Tajik	Kyrgyz	Total
Average Age	28.6	24.8	26.7
**Marital Status**			
Single	10 (66%)	8 (53%)	18 (60%)
Widowed	3 (20%)	7 (47%)	10 (33%)
Divorced	2 (13%)	0 (0%)	2 (7%)
**Children**			
0	8 (53%)	8 (53%)	16 (53%)
1	5 (33%)	4 (27%)	9 (30%)
2	1 (7%)	2 (13%)	3 (10%)
Pregnant	1 (7%)	1 (7%)	2 (7%)
**Education**			
Secondary	8 (53%)	6 (40%)	14 (47%)
Some College	3 (20%)	3 (20%)	6 (20%)
College	4 (27%)	5 (33%)	9 (30%)
Trade School	0 (0%)	1 (7%)	1 (3%)
**Employment**			
Seller	6 (40%)	1 (7%)	7 (23%)
Cleaner	4 (27%)	7 (47%)	11 (37%)
Sex Work	1 (7%)	1 (7%)	2 (7%)
Other	0 (0%)	3 (20%)	3 (10%)
Unemployed	4 (27%)	3 (20%)	7 (23%)
Total	15	15	30

## References

[1] Oishi N (2005). Women in motion: Globalization, state policies, and labor migration in Asia.

[2] UNIFEM (2005). Estimate of needs and necessities of female labor migrants: Central Asia and Russia.

[3] Karimov A, Maksudov M (2010). Economic crisis forcing Central Asian migrants to leave Russia.

[4] Kramer MA, Van Veen MG, De Coul E, Geskus RB, Coutinho RA, Van De Laar MJ, Prins M (2008). Migrants travelling to their country of origin: A bridge population for HIV transmission?. Sex Transm Infect.

[5] Marmot M, Friel S, Bell R, Houweling TA, Taylor S (2008). Closing the gap in a generation: Health equity through action on the social determinants of health. Lancet.

[6] Magis-Rodríguez C, Lemp G, Hernandez MT, Sanchez MA, Estrada F, Bravo-García E (2009). Going North: Mexican migrants and their vulnerability to HIV. J Acquir Immune Defic Syndr.

[7] Mmbaga EJ, Leyna GH, Hussain A, Mnyika KS, Sam NE, Klepp KI (2008). The role of in-migrants in the increasing rural HIV-1 epidemic: Results from a village population survey in the Kilimanjaro region of Tanzania. Int J of Infect Dis, official publication of the International Society for Infectious Diseases.

[8] Puri M, Cleland J (2006). Sexual behavior and perceived risk of HIV/AIDS among young migrant factory workers in Nepal. J Adolesc Health.

[9] Yang X, Derlega VJ, Luo H (2007). Migration, behaviour change and HIV/STD risks in China. AIDS Care.

[10] O’Conner W (2009). Russia: Central Asia’s female labor migrants grapple with uncertainty.

[11] Weine SM, Bahromov M, Mirzoev A (2008). Unprotected Tajik male migrant workers in Moscow at risk for HIV/AIDS. Journal of Immigrant Minority Health.

[12] Hong Y, Li X, Yang H, Fang X, Zhao R (2009). HIV/AIDS-related sexual risks and migratory status among female sex workers in a rural Chinese county. AIDS Care.

[13] Ojeda VD, Strathdee SA, Lozada R, Rusch ML, Fraga M (2009). Associations between migrant status and sexually transmitted infections among female sex workers in Tijuana, Mexico. Sexually Transmitted Infection.

[14] Zuma K, Gouws E, Williams B, Lurie M (2003). Risk factors for HIV infection among women in Carletonville, South Africa: Migration, demography and sexually transmitted diseases. International Journal of STD AIDS.

[15] Kossoudji SA, Ranney SI (1984). The labor market experience of female migrants: the case of temporary Mexican migration to the U.S. The International Migration Review.

[16] Bronfman MN, Leyva R, Negroni MJ, Rueda CM (2002). Mobile populations and HIV/AIDS in Central America and Mexico: Research for action. AIDS.

[17] Singh G (2007). Paradoxical payoffs: Migrant women, informal sector work, and HIV/AIDS in South Africa. New Solutions a Journal of Environmental and Occupational Health Policy.

[18] Yang X, Xia G (2006). Gender, migration, risky sex, and HIV infection in China. Studies in Family Planning.

[19] Bandyopadhyay M, Thomas J (2002). Women migrant workers’ vulnerability to HIV infection in Hong Kong. AIDS Care.

[20] Cash K, Anansuchatkul B, Busayawong W (1999). Understanding the psychosocial aspects of HIV / AIDS prevention for northern Thai single adolescent migratory women workers. Appl Psychol.

[21] Ford K, King G, Nerenberg L, Rojo C (2001). Aids knowledge and risk behaviors among Midwest migrant farm workers. AIDS Educ and Prev.

[22] Hirsch JS, Higgins J, Bentley ME, Nathanson CA (2012). The social constructions of sexuality: Marital infidelity and sexually transmitted disease-HIV risk in a Mexican migrant community. Am J Public Health.

[23] Islam MM, Conigrave KM, Miah MS, Kalam KA (2010). HIV awareness of outgoing female migrant workers of Bangladesh: A pilot study. J Immigr Minor Health.

[24] Organista KC, Organista PB, Garcia De Alba JE, Castillo Moran MA, Carrillo H (1996). AIDS and condom-related knowledge, beliefs, and behaviors in Mexican migrant laborers. Hisp J Behav Sci.

[25] Kogan SM, Brody GH, Chen Y, Grange CM, Slater LM, DiClemente RJ (2010). Risk and protective factors for unprotected intercourse among rural African American young adults. Public Health Rep.

[26] Mueller T, Gavin L, Oman R, Vesely S, Aspy C, Tolma E, Rodine S (2010). Youth assets and sexual risk behavior: Differences between male and female adolescents. Health Educ Behav.

[27] Sychareun V, Thomsen S, Faxelid E (2011). Concurrent multiple health risk behaviors among adolescents in Luangnamtha province, Lao PDR. BMC Public Health.

[28] Li N, Boulay M (2010). Individual, familial and extra-familial factors associated with premarital sex among Bangladeshi male adolescents. Sex Health.

[29] World Health Organization (2008). Epidemiological Fact Sheet on HIV/AIDS 2008.

[30] Weine SM, Kashuba AB (2012). Labor Migration and HIV Risk: A Systematic Review of the Literature. AIDS Behav.

[31] Sandelowski M (2000). Whatever happened to qualitative description?. Res Nurs Health.

[32] Corbin J, Strauss A (2008). Basics of qualitative research: Techniques and procedures for developing grounded theory.

[33] Muhr T (2004). ATLAS/ti 5.0 user’s manual and reference. Version 5.0.

[34] Miles MB, Huberman AM (1994). Qualitative data analysis: An expanded sourcebook.

[35] Tilden VP, Weinert C (1987). Social support and the chronically ill individual. Nurs Clin North Am.

[36] Russian Federal Migration Services (2010). Paperwork.

[37] Hirsch JS, Wardlow H, Smith DJ, Phinney H (2010). The secret: Love, marriage, and HIV.

[38] United Nations Development Fund for Women (UNIFEM) (2001). Turning the tide, CEDAW and the gender dimensions of the HIV/AIDS pandemic.

[39] Busza J, Baker S (2004). Protection and participation: an interactive programme introducing the female condom to migrant sex workers in Cambodia. AIDS Care.

